# Al_0.5_CoCrFeNi_2_ High Entropy Alloy Particle Reinforced AZ91 Magnesium Alloy-Based Composite Processed by Spark Plasma Sintering

**DOI:** 10.3390/ma14216520

**Published:** 2021-10-29

**Authors:** Chun Chiu, Hsun-Hsiang Chang

**Affiliations:** Department of Mechanical Engineering, National Taiwan University of Science and Technology, Taipei 106335, Taiwan; m10703517@mail.ntust.edu.tw

**Keywords:** AZ91 magnesium alloy, high-entropy alloy, metal matrix composite, spark plasma sintering

## Abstract

In this study, AZ91 magnesium-alloy-based metal matrix composites (MMCs) reinforced with 10 wt% of Al_0.5_CoCrFeNi_2_ high-entropy alloy (HEA) particles and SiC particles were prepared by a spark plasma sintering (SPS) process at 300 °C. The effects of reinforcements on the microstructure and mechanical properties of AZ91-based MMCs were studied. The results showed that AZ91–HEA composite consisted of α-Mg, Mg_17_Al_12_ and FCC phases. No interfacial reaction layer was observed between HEA particles and the Mg matrix. After adding HEA into AZ91, the compressive yield strength (C.Y.S) of the AZ91–HEA composite increased by 17% without degradation of failure strain. In addition, the increment in C.Y.S brought by HEA was comparable to that contributed by commonly used SiC reinforcement (15%). A relatively low porosity in the composite and enhanced interfacial bonding between the α-Mg matrix and HEA particles make HEA a potential reinforcement material in MMCs.

## 1. Introduction

The goal for energy saving through improving fuel efficiency has driven the need for lightweight materials. Magnesium alloy is one of the most important lightweight materials in the automotive, aerospace and biomaterial industries and has attracted much attention because of its high specific strength to weight ratio and recyclability [1,2,3,4]. However, the strength of conventional AZ (Mg–Al–Zn) and AM (Mg–Al–Mn) series Mg alloys will not meet the expanded requirements for load-bearing components with the rapid development of science and technology [5]. As a result, research into high-performance Mg alloys that will satisfy the extended industrial applications is essential.

One way to strengthen Mg alloys is by adding reinforcement phase into Mg matrix and making Mg alloy-based composites. Ceramic hard particles, such as Al_2_O_3_, ZnO, SiC, TiC and Y_2_O_3_, have been widely studied for application in Mg-based composites. However, the difference of thermal expansion coefficient, poor wettability and interfacial reaction have hindered their application [6]. To solve the issues, metallic glass (MG) phase reinforcement has been studied. MGs have superior elastic properties, strength and most importantly, good wettability with metal matrix (due to its metallic nature) [7]. A metallic-glass-reinforced AZ91 magnesium-alloy-based composite was studied and displayed improved hardness and strength compared to the AZ91 alloy [8].

Recently, an emerging category of alloys, high-entropy alloys (HEAs), has attracted lots of attention. Unlike conventional alloys, HEAs consist of five or more principal elements, and each element’s concentration is from 5 to 35 at% [9]. Because of the four essential effects, such as high-entropy effect, sluggish diffusion effect, severe lattice distortion and cocktail effect, HEAs possess high strength, excellent corrosion resistance and wear resistance. In addition to traditional structural applications, potential applications of HEAs can be found in surface coating, environmental protection, gas sensing, energy storage and waste heat recovery [10,11]. HEAs also have good wettability with metal and thermal expansion coefficients similar to those of metals. Thus, it has potential for the application as a reinforcement in Mg-alloy-based metal matrix composites and can solve the compatibility issue between metal matrices and ceramic reinforcements. HEAs can be classified into 3D transition metal HEAs, 4f transition metal HEAs, refractory metal HEAs and light metal HEAs [10]. Among different series of HEAs, the one based on principle elements of Al, Co, Cr, Fe and Ni has been extensively studied for its microstructure, phase stability and mechanical properties [9,10,11,12,13,14,15,16]. Equiatomic AlCoCrFeNi HEA is a dual-phase (FCC and BCC) material, and it undergoes a phase transformation at high temperature [17]. On the other hand, Al_0.5_CoCrFeNi_2_ [18,19], which has an FCC structure, exists a high thermal stability and is more suitable for application as a reinforcement in MMCs. 

The fabrication of Mg-based metal matrix composites (MMCs) has been carried out by stir casting or by the powder metallurgy (P/M) method, which combines powder preparation and consolidation of powders. Unlike the agglomeration of reinforced particles in the ingot prepared by stir casting, uniform distribution of reinforcements is obtained in the samples prepared by the P/M method. Recently, spark plasma sintering (SPS) has been applied in P/M. Compared to traditional hot pressing method, samples processed by SPS have high density and less grain or microconstituent growth due to the short holding time. Studies of Mg and its alloys prepared by SPS have been reported, and the results indicated that the strength of the Mg alloy prepared by SPS is superior to that of the cast one [20,21,22].

In the present work, we explored a new way of strengthening of Mg alloy by addition of HEA particles. The Al_0.5_CoCrFeNi_2_ HEA was chosen as the reinforcement due to its high strength and its well-known properties, which make it suitable for the early-stage development of HEA-reinforced MMCs. Al_0.5_CoCrFeNi_2_-reinforced AZ91 composites were fabricated by SPS, and their microstructures and mechanical properties were studied. SiC-reinforced AZ91 composites were also prepared for comparison of their mechanical properties.

## 2. Materials and Methods

Atomized AZ91 powder with an average particle size of 45 μm was purchased from Weihao Magnesium Powder Co., Ltd. (Tangshan, China). Al_0.5_CoCrFeNi_2_ (at%) high entropy alloy (HEA) powder fabricated by gas-atomization process was supplied by Nano Manufacturing and Surface Treatment Lab, NTUST, Taipei, Taiwan (R.O.C.). Details of the HEA powder preparation can be found in reference [23]. The average particle size of HEA powder was 28 μm. SiC powder with an average particle size of 38 μm was acquired from Taicheng Metallic Materials Co. Ltd. (Wuxi, China).

Powder mixtures of AZ91 with 10 wt% of HEA and SiC powder were blended in a twin arm shaker for 5 min. All of the powder handling was implemented in a glove box filled with purified argon to prevent oxidation. Subsequently, AZ91–HEA and AZ91–SiC powder mixtures were consolidated by spark plasma sintering (SPS). The SPS was carried out under vacuum in a SPS-515S system (SPS SYNTEX INC, Tokyo, Japan,) with a sintering temperature of 300 °C, a pressure of 50 MPa and a holding time of 5 min. The nomenclature and processing of the alloys and composites in the present study are summarized in Table 1. The porosity (ϕ) of the sample was calculated from the difference between the theoretical (ρ_T_) and experimental density (ρ_E_):

ϕ = 1 − ρ_E_/ρ_T_ (1)

Microstructural characterization was conducted with a scanning electron microscope (SEM) (JEOL JSM-6500F, Tokyo, Japan) equipped with Energy Dispersive Spectroscopy (EDS). Phase analysis was performed by X-ray diffraction (XRD) (Bruker D2, Billerica, MA, USA) using Cu Kα radiation. The diffraction data were collected with a step size of 0.02° and time of 0.5 s in the scan range of 20 to 80°. Dislocation densities of AZ91, AZ91–HEA and AZ91–SiC composites were calculated by analyzing XRD patterns using the Williamson–Hall equation [24] and Williamson–Smallman equation [25]:

(2) $$\frac{\beta \cos\theta }{\lambda }=\frac{1}{D_{v}}+2\varepsilon \left(\frac{2\sin\theta }{\lambda }\right)$$

where D_v_ is the average crystallite size, ε is the microstrain, b is the integral breadth, λ is the wave-length and θ is the position of the analyzed peak maximum. Dislocation density (ρ) is related to microstrain and can be calculated using the following equation:

(3) $$\rho =\frac{k\varepsilon }{b^{2}}$$

where k is the material constant (considered as 1 for Mg alloy), and b is the magnitude of Burger’s vector.

Mechanical properties of the composites were evaluated by using compression test, microhardness test and nanoindentation test. Compression test was performed at room temperature using a universal material testing machine MTS810 (MTS, Eden Prairie, OR, USA). Samples with a diameter of 10 mm and a height of 10 mm were tested under a strain rate of 10^−3^/s following ASTM standard E9-89a. Three tests were performed for each alloy and composite to obtain average value and standard deviation. The strain hardening rate (θ) was calculated using the following equation:

θ = dσ_t_/dε_t_ (4)

where σ_t_ and ε_t_ are true stress and true strain, respectively.

Vickers microhardness tests were performed using Akashi MVK-H1 (Mitutoyo, Kawasaki, Japan) microhardness tester. The load and the dwell time were 200 g and 15 s, respectively. The spacing between indents was at least three times the diagonal. Nanoindentation tests were performed by using TI-900 Nanoindentator (TriboIndenter, Hysitron, Billerica, MA, USA). The maximum load was set as 1000 μN. The load function for nanoindentation is of 5 × 5 × 5 mode, which means taking 5 s to increase the load to 1000 μN, holding for 5 s and then reducing to zero in another 5 s. Ten measurements were carried out for hardness tests, and an average value was presented.

## 3. Results

### 3.1. Microstructural Characterization

#### 3.1.1. Characterization of AZ91, HEA and SiC Powders

**Figure 1:**
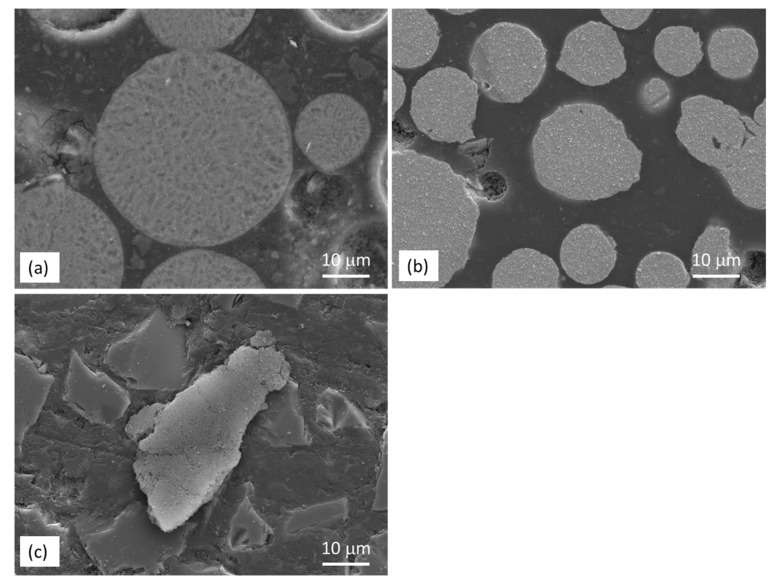
SEM micrographs showing the cross section of (**a**) AZ91, (**b**) HEA and (**c**) SiC powders.

**Figure 2:**
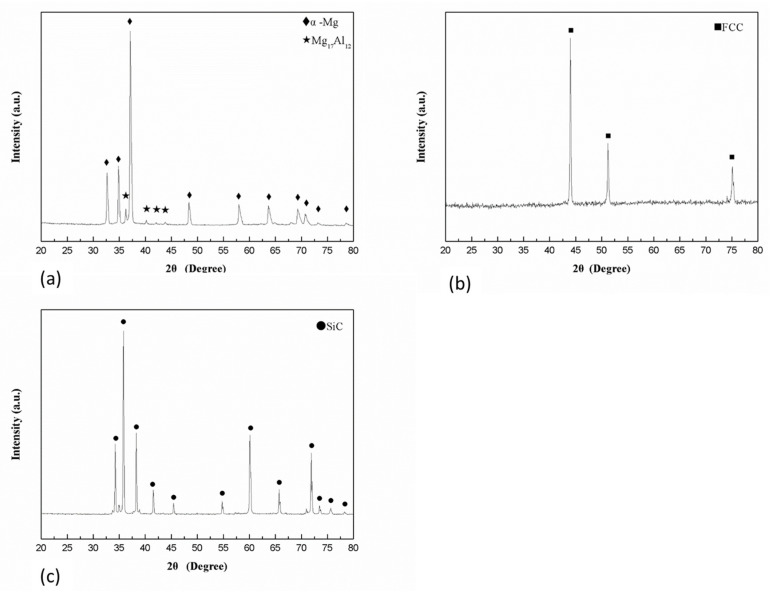
XRD patterns of (**a**) AZ91, (**b**) HEA and (**c**) SiC powders.

SEM micrographs of the cross section of AZ91, HEA (Al_0.5_CoCrFeNi_2_) and SiC powders are shown in Figure 1, and the corresponding XRD patterns are given in Figure 2. The gas-atomized AZ91 and HEA powders had a circular cross section and the SiC powder had an irregular shape. AZ91 powder contained α-Mg and Mg_17_Al_12_ precipitates along the grain boundary. Diffraction peaks at 43.5, 51.2 and 75.0° were observed in the XRD pattern of HEA powder, indicating that HEA has an FCC structure. No precipitates were observed in HEA powder. Only the SiC phase was observed in as-received SiC powders.

#### 3.1.2. Characterization of SPS Samples

**Figure 3:**
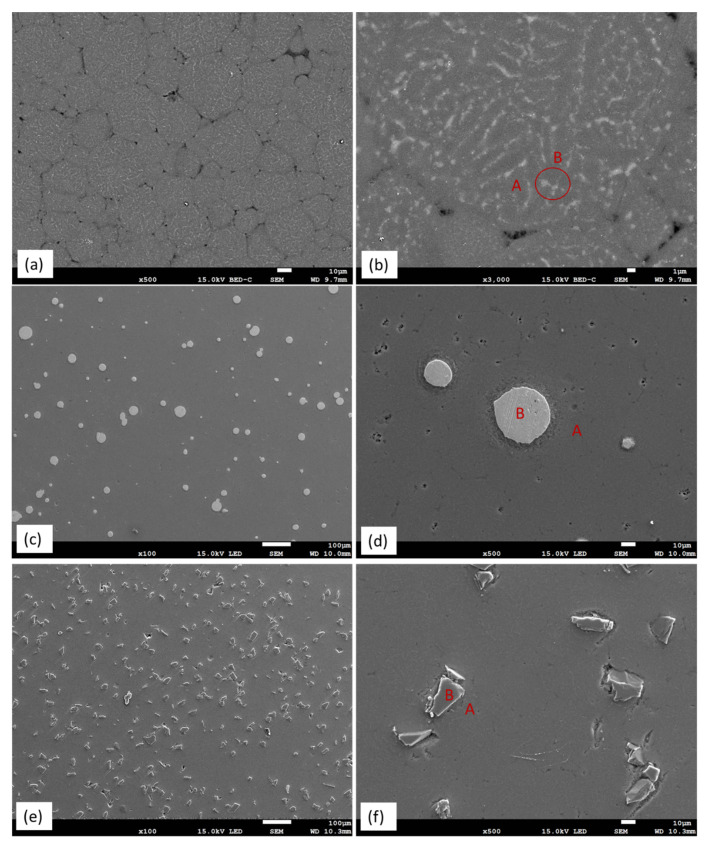
SEM micrograph showing (**a**) particle boundaries in AZ91, (**b**) precipitates in Mg matrix in AZ91, (**c**) distribution of HEA in AZ91–HEA composite, (**d**) boundary of HEA particle in AZ91 matrix, (**e**) distribution of SiC in AZ91–SiC composite and (**f**) boundary of SiC particle in AZ91 matrix (EDS analysis results of area A (matrix) and B (reinforcement) are given in Table 2).

**Figure 4:**
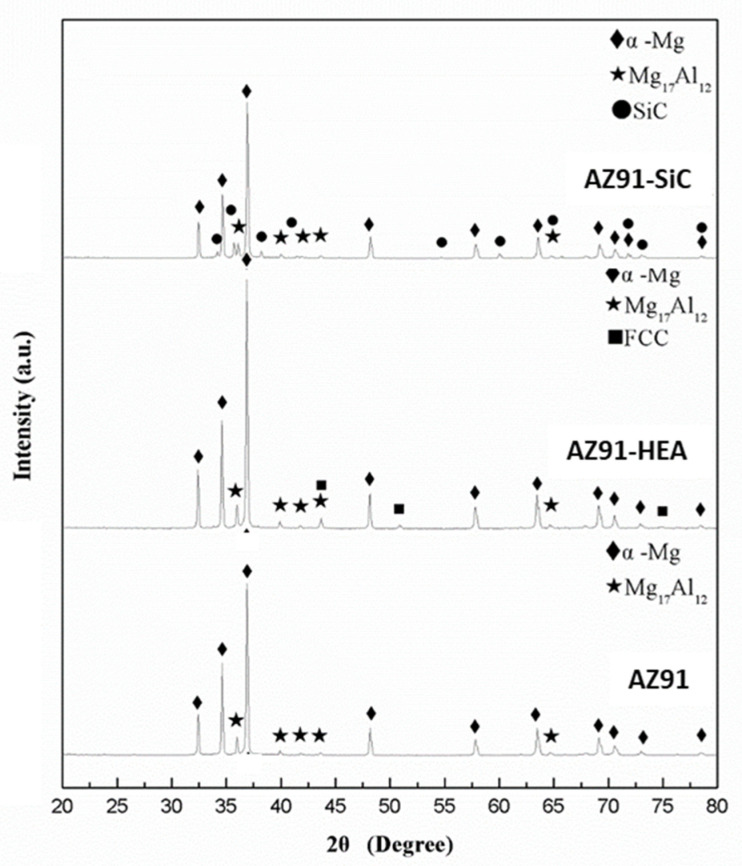
XRD patterns of AZ91, AZ91–HEA and AZ91–SiC samples.

Figure 3a shows the SEM micrograph of the sintered AZ91 sample. Pores could be seen along the particle boundary and the porosity measured by image analysis was ~1.0%. A higher magnification micrograph (Figure 3b) illustrates the microstructure of the sintered AZ91 consisting of brighter precipitates (area B) located along the boundary of gray grains (area A). By combing results from EDS and XRD analysis (Table 2 and Figure 4), the brighter precipitate was identified as the Mg_17_Al_12_ phase, and the gray grain was α-Mg. The results indicated that no phase transformation occurred in the AZ91 sample after the SPS process, and the precipitates still distributed along the grain boundary.

Figure 3c shows the microstructure of the sintered AZ91–HEA composite. HEA particles distributed uniformly in the AZ91 matrix. A clear boundary between HEA particle and matrix could be seen and no interfacial reaction layer was observed (Figure 3d). The porosity in the sintered composite was 0.8%. EDS analysis results indicated that compositions of α-Mg grain (area A) and HEA (area B) were almost identical to that of α-Mg in sintered AZ91 and the composition of as-received HEA, respectively. The observation that no phase transformation occurred in the AZ91–HEA composite after SPS process was also confirmed by XRD analysis (Figure 4). HEA had a single FCC phase after SPS, and α-Mg with Mg_17_Al_12_ was still detected. Moreover, no interfacial reaction layer was observed implying there was no interdiffusion between reinforcement and matrix.

The microstructure of sintered AZ91–SiC composite showed uniformly distributed SiC particles in the AZ91 matrix (Figure 3e). No interface reaction layer was formed between reinforcement and the matrix after SPS. Combined with the results from the EDS and XRD analyses, α-Mg, Mg_17_Al_12_ and SiC were the only phases presented in the composite. No phase transformation occurred after SPS. However, the porosity of the sintered sample was 3.8%, which was four times higher than those from the sintered AZ91 and the AZ91–HEA composite.

**Figure 5:**
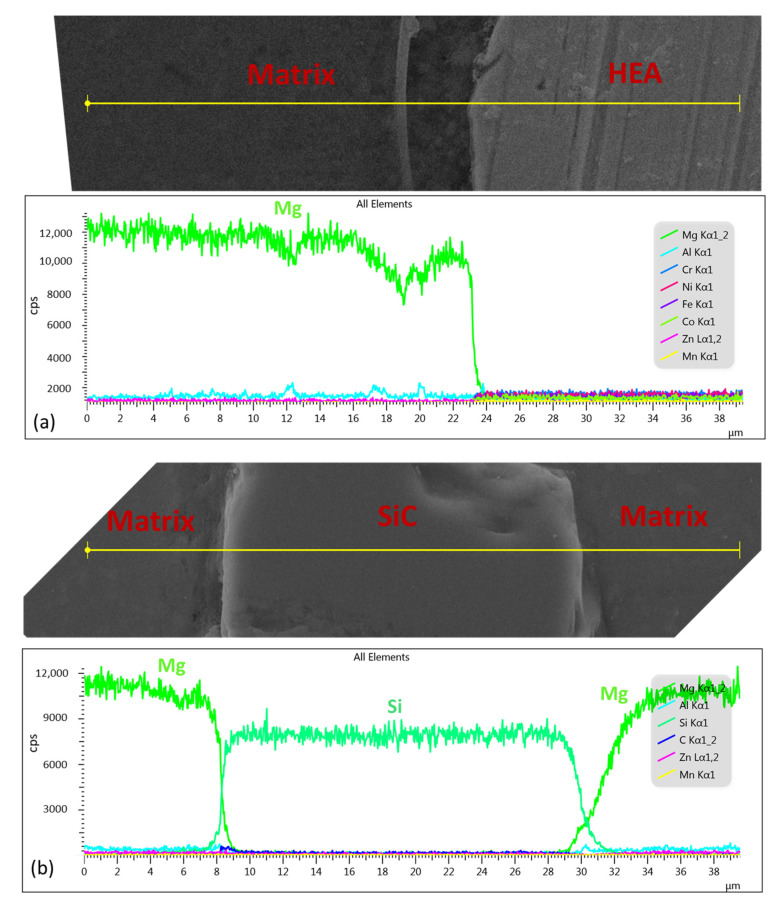
EDS line scan of (**a**) HEA and (**b**) SiC in the AZ91-based composites.

From the SEM images in Figure 3, a clear interface between the matrix and the reinforced phase HEA and SiC was observed. The compositions of the α-Mg matrix in the composites were similar to that in the AZ91 (Table 2). The composition of HEA in the sintered composite had no obvious change, compared to composition of as-received HEA powder. Moreover, from the results of the EDS line scans of HEA and SiC particles in the composites (Figure 5), no interfacial reaction layer was formed between the HEA, SiC and Mg matrix. The above results confirmed that no diffusion between the reinforced particle and the matrix occurred during the SPS process. No aggregations of reinforced particles were observed in the AZ91–HEA and AZ91–SiC composites.

### 3.2. Mechanical Properties of SPS Samples

**Figure 6:**
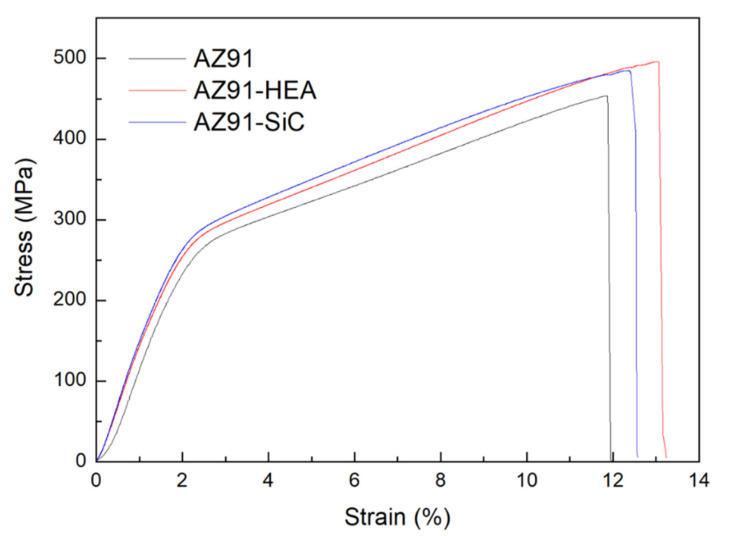
Engineering compressive stress-strain curves of AZ91 alloy, AZ91–HEA and AZ91–SiC composites.

**Figure 7:**
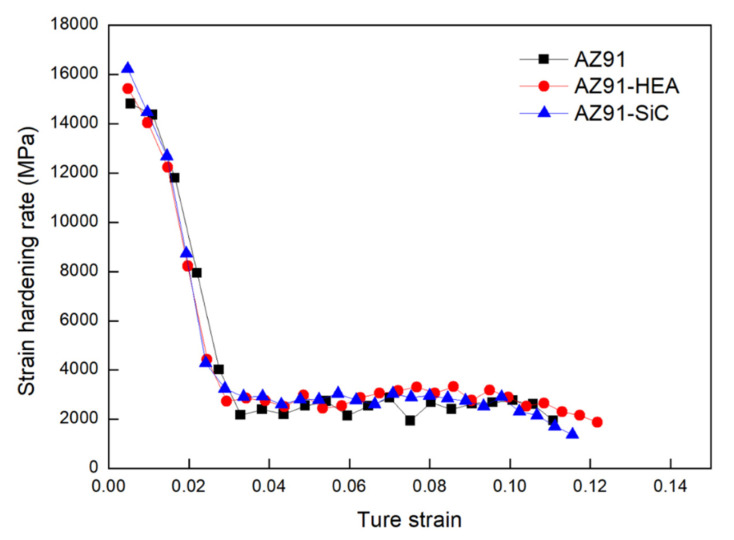
Curves of strain hardening rate against true strain for AZ91 alloy, AZ91–HEA and AZ91–SiC composites.

The results of engineering compressive stress-strain curves of the AZ91 alloy and the AZ91–HEA and AZ91–SiC composites are shown in Figure 6 (Enlarged sections of curves of the composites are shown in Figures S1 and S2 in Supplementary Material). The enhancement of compressive yield strength (C.Y.S), ultimate compressive strength (U.C.S.) of the AZ91 composites can be observed when compared to those of the AZ91 alloy. Figure 7 shows the strain hardening rate versus the true strain for various samples. Higher strain hardening rates were found in the AZ91 alloys reinforced with SiC and HEA. For all of the samples, the hardening occurred at the initial stage of the deformation. All samples displayed similar decreasing of work hardening tendency, indicating that behavior of plastic deformation of the alloy and composites is almost the same.

Mechanical properties of the sintered samples are summarized in Table 3. From the summarized results of compression tests, the effect of reinforcement on the strength of composite could be observed. It could be seen that the hardness and compressive yield strength (C.Y.S) of AZ91 increased after adding 10 wt% of reinforcement. The hardness first increased from 93 HV in AZ91 to 123 HV in the AZ91–SiC composite and further increased to 138 HV in the AZ91–HEA composite. The C.Y.S increased from 178 MPa in AZ91 to 204 and to 209 MPa in AZ91–SiC and AZ91–HEA, respectively. No degradation of the failure strain was observed after the addition of HEA and SiC. The failure strain of the AZ91–SiC was similar to that of AZ91, and the failure strain of AZ91–HEA increased slightly from 12.2% to 13.7%.

## 4. Discussion

AZ91-based metal matrix composites reinforced by high-entropy alloys (HEA) and SiC particles were synthesized in the present study by the spark plasma sintering (SPS) process. The results from the strain hardening rate calculation show that the AZ91–HEA and AZ91–SiC composites exhibited higher strain hardening rates when compared to unreinforced AZ91 alloy (Figure 7). The dislocation densities of AZ91 and the AZ91–HEA and AZ91–SiC composites were calculated by analyzing XRD patterns and were determined to be ~3.9 × 10^15^ m^−2^ for all of the samples. The result showed that no huge difference was observed in the dislocation densities of the sintered samples, and the higher strain hardening rates in the composites were related to the reaction between reinforcements (HEA and SiC) and dislocations. The ability of HEA and SiC to increase the strain hardening rate may be attributed to the capacity to impede dislocation motion, which was also observed in other Mg-based composites reinforced with ceramic particles [26]. Moreover, other strengthening mechanisms may also contribute to the enhancement of the hardening rate.

The effect of HEA and SiC particles on the mechanical properties of the sintered composites could be discussed in terms of different strengthening mechanisms including grain boundary strengthening, Orowan strengthening, thermal mismatch strengthening, load transfer and solid solution strengthening.

The increase in the yield strength due to the refinement of the grain size of the matrix in the composites can be estimated using the following equation derived from the Hall–Petch equation [27,28]:

(5) $$\Delta \sigma_{\mathrm{Hall}-\mathrm{Petch}}=k\left(d_{\mathrm{com}}^{-\frac{1}{2}}-d_{\mathrm{AZ}91}^{-\frac{1}{2}}\right)$$

where k is the Hall–Petch coefficient (0.27 MPam^−1/2^ [29]), d_com_ and d_AZ91_ are the average grain size of Mg in the composite material and AZ91, respectively. The grain sizes of Mg in the sintered AZ91, AZ91–HEA and AZ91–SiC were 5.4, 5.3 and 5.1 μm, respectively. Only marginal changes in grain size were observed after adding HEA or SiC particles. From Equation (5), the increased yield strengths due to grain refinement in the AZ91–HEA and AZ91–SiC composites were 1 and 4 MPa, respectively. The addition of HEA and SiC particles in the composites did not cause a significant grain refinement and led to a slight increase in yield strength.

In the current study, AZ91 rather than pure Mg was used for preparing composites, implying that the matrix contained α-Mg solid solution rather than pure Mg. As a result, the effect of solid solution strengthening should be examined. Impurity atoms in solid solution can impede the movement of dislocation and increase the yield strength of the base metal. The increase in yield strength due to solid solution strengthening can be estimated using the following equation [30]:

(6) $$\Delta \sigma_{\mathrm{ss}}=C(X_{\mathrm{com}}^{2/3}-X_{\mathrm{AZ}91}^{2/3})$$

where C equals 197 MPa [30]. X_com_ and X_AZ91_ are the atomic percent (at%) of impurity atom in α-Mg in the composite and AZ91, respectively. The concentration of Al in Mg solid solution in AZ91, AZ91–HEA and AZ91–SiC were 7.31, 7.39 and 7.35at%, respectively. From Equation (6), Δσ_ss_ in AZ91–HEA was 0.3 MPa, while that in AZ91–SiC was 0.1 MPa. The effect of solid solution strengthening led to a slightly increase in yield strength.

The contribution of Orowan strengthening to the increment in yield strength is due to the interaction of precipitates or reinforcements with dislocations, which can be calculated using the following equations [26]:

(7) $$\Delta \sigma_{\mathrm{Orowan}}=0.5\frac{\mathrm{Gb}}{\pi \lambda \sqrt{1-\nu }}\ln\frac{d}{b}$$

(8) $$\lambda =d\left(\sqrt{\pi /4f}-1\right)$$

where G is the shear modulus of the matrix (16.5 GPa [31]), b is the value of the Burgers vector of the matrix (0.32 nm [30]), d is the average diameter of the precipitate or reinforced particles (28 μm for HEA and 38 μm for SiC), λ is the spacing between particles and f is the volume fraction of precipitate or reinforcement. In the AZ91-based composite, both f the precipitation of Mg_17_Al_12_ and the distribution of reinforcements such as HEA and SiC particles could contribute to the increasing yield strength. The addition of HEA and SiC into AZ91 did not change the volume fraction and size of Mg_17_Al_12_ significantly. The volume fractions of Mg_17_Al_12_ in the AZ91, AZ91–HEA and AZ91–SiC were 10, 10.2 and 11.7%, respectively. The particle size of Mg_17_Al_12_ is ~0.5 μm in all of the samples. The increasing yield strength was 8 MPa in the AZ91 alloy. After adding HEA and SiC, the values were 8.3 and 9.2 MPa, respectively. The results indicated that the contribution of Mg_17_Al_12_ on the enhancement of yield strength was similar in the AZ91 alloy and the composites. As a result, its effect could be neglected when calculating the difference between the yield strength of AZ91 and AZ91-based composites.

Regarding the strengthening due to the dispersion of HEA and SiC particles, the values were 0.10 and 0.12 MPa, respectively, indicating that the Orowan strengthening due to the dispersion of HEA and SiC particles on AZ91 matrix was not the major contributor for the increment in yield strength in the AZ91–HEA and AZ91–SiC composites. It was reported that the size of the reinforced particle had to be less than 1 μm to obtain a strong pinning effect of dislocation [32]. The coarse particle size of HEA and SiC resulted in a weak pinning effect in Orowan strengthening. The particle size of HEA and SiC has to be further refined to enhance the Orowan strengthening effect.

The thermal mismatch will lead to the generation of dislocation in the area surrounding the reinforced particles upon cooling after the SPS process and eventually increases the yield strength. The enhancement of yield strength due to the mismatch of CTE can be estimated using the following equation [33,34]:

(9) $$\Delta \sigma_{\mathrm{CTE}}=\sqrt{3}\beta \mathrm{Gb}\sqrt{\frac{12f_{r}\Delta \alpha \Delta T}{\left(1-f_{r}\right)\mathrm{bd}}}$$

where β is the strengthening coefficient (1.25 [34]), G is the shear modulus of matrix, b is the Burgers vector, f_r_ is the volume fraction of reinforcement, Δα is the difference between the coefficient of thermal expansion between the matrix and the reinforcement, ΔT is the difference between the processing and testing temperatures and d is the diameter of the reinforced particles.

The coefficient of thermal expansion (CTE) of AZ91, HEA and SiC are 29 × 10^−6^/K, 10 × 10^−6^/K and 4.7 × 10^−6^/K, respectively. Clearly, there exists a difference between the CTE of the AZ91 matrix and the reinforcements (HEA and SiC). The increment in yield strength due to thermal mismatch in the AZ91–HEA was 10 MPa, while the increment in the AZ9–1SiC was 9.9 MPa.

In a composite material, load can be transferred from the matrix to the reinforcement particles, leading to the enhancement of yield strength. The increment in yield strength as a result of load transfer can be expressed as [34]:

(10) $$\Delta \sigma_{\mathrm{Load}}=0.5f_{r}\sigma_{m}$$

where σ_m_ is the yield strength of the AZ91 alloy, and f_r_ is the volume fraction of reinforcement. The estimated increments in yield strength in the AZ91–HEA and AZ91–SiC composite were 3 and 6 MPa, respectively.

**Figure 8:**
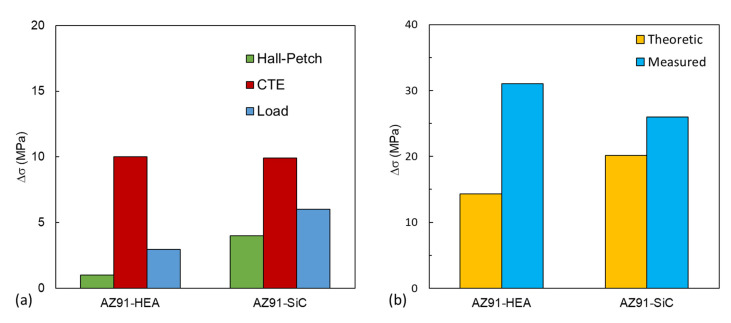
(**a**) The increment in compressive yield strength due to the contribution of grain-size strengthening, dislocation strengthening and effect of load transfer from matrix to particle in the AZ91–HEA and AZ91–SiC composites. (**b**) Theoretical and measured values of the increased yield strength in the AZ91–HEA and AZ91–SiC composites.

The increment in yield strength caused by different strengthening mechanisms is summarized in Table 4 and plotted in Figure 8a. In the AZ91–HEA composite, the thermal mismatch effect contributed the most (69%) to the increment in yield strength, followed by the load transfer effect (21%), the grain-refinement effect (Hall–Petch) (7%) and the Orowan effect (0.7%). A similar trend was also observed in the AZ91–SiC composite.

Assuming all of the enhancement brought by various strengthening mechanisms could be added up linearly [31,35], the total increment in yield strength of AZ91-based composite estimated by different strengthening mechanisms can be expressed as:

(11) $$\Delta \sigma_{\mathrm{theoretic}}=\Delta \sigma_{\mathrm{CTE}}+\Delta \sigma_{\mathrm{Load}}+\Delta \sigma_{\mathrm{Hall}-\mathrm{Petch}}+\Delta \sigma_{\mathrm{Orowan}}+\Delta \sigma_{\mathrm{ss}}$$

As shown in Figure 8b, theoretical values of increment in yield strength in AZ91–HEA and AZ91–SiC composites were 14 and 20 MPa, respectively. The measured values obtained from compression tests for AZ91–HEA and AZ91–SiC were 31 and 26 MPa, respectively. The increment in yield strength could be roughly estimated using strengthening mechanisms.

According to the theoretical calculation, the addition of 10 wt% of SiC in AZ91 should have a slightly higher strengthening effect. However, the weakening effect due to higher porosity and weaker bonding between the matrix and reinforcement in AZ91–SiC composite was not taken into account in the calculation. As shown in Figure 3f, debonding was observed in the interface between the AZ91 matrix and SiC particles, implying a weaker interface bonding. The porosity in the AZ91–SiC (3.8%) is higher than that in AZ91–HEA (0.8%) and reduced the strength of the AZ91–SiC composite. It was also observed that the theoretical strength was lower than the measured strength. The deviation may be attributed to the approximate parameters used for HEA, nonuniform particle size and the assumption that irregular particles could be treated as spherical particles.

**Figure 9:**
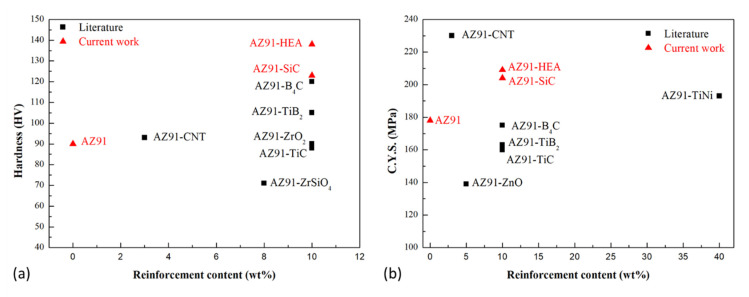
(**a**) Hardness and (**b**) compressive yield strength (C.Y.S.) of AZ91 alloy and AZ91-based metal matrix composites prepared by powder metallurgy route in the current work and from literatures.

Hardness and compressive yield strength of AZ91-based metal matrix composites prepared by the powder metallurgy route in the current work and from the literature are shown in Figure 9 [35,36,37,38,39,40]. It can be seen that the hardness and strength of AZ91–HEA composite synthesized in the current work was superior to those in the AZ91-based composites reinforced with oxide, nitride and boride. The harnesses of the AZ91 matrix and HEA-reinforced particles were 84 and 208 HV, respectively. In addition to HEA’s higher hardness value compared to the matrix, its metallic nature, which resulted in a better interface bonding between the matrix and reinforcement (Figure 3e), also made HEA a promising reinforcement for the metal matrix composite.

In the present study, we proposed a new approach by reinforcing AZ91 alloys with HEA particles. Preliminary results showed that the HEA particle has potential to be used for reinforcement in metal matrix composites. The analysis performed indicated that the particle size of HEA reinforcement should be refined to enhance its contribution of hardening effect of HEA. The effects of particle size, fraction of HEA and post-sintering extrusion on strength will be the subject of our future study.

## 5. Conclusions

In the current work, AZ91 metal matrix composites reinforced with 10 wt% of HEA particles were fabricated by spark plasma sintering process. AZ91–SiC composites were also synthesized for comparison of their mechanical properties. Microstructure and mechanical properties of the sintered composites were studied and the following conclusions could be drawn:

1. AZ91–HEA composite with a uniform distribution of HEA particles in the matrix could be manufactured using spark plasma sintering process. The composite consisted of α-Mg, Mg_17_Al_12_ and FCC phase. Phase transformation was not observed in the sintered composite compared to the as-milled powder. No reaction layer was formed in the interfacial region between HEA and α-Mg matrix.
2. The addition of HEA efficiently enhances hardness and compressive yield strength (C.Y.S) of AZ91–HEA. After adding HEA particles, hardness and C.Y.S of the composite were increased by 48% and 17%, respectively.
3. In AZ91–HEA composite, thermal mismatch effect, load transfer effect and grain-refinement effect contributed to the increment in yield strength. Among them, the thermal mismatch effect was the major factor.
4. AZ91–HEA displayed a slightly higher C.Y.S (209 MPa) than that of AZ91–SiC (204 MPa). The strengthening effect of HEA was comparable to that of the commonly used SiC reinforcement in metal matrix composite. The reason was due to the less porosity and enhanced interfacial bonding between α-Mg matrix and HEA particles.

## Figures and Tables

**Table 1:** Nomenclature and processing of the alloys and composites studied in the present study.

Sample ID	Processing
AZ91	AZ91 powder compacted by SPS
AZ91–HEA	AZ91-10 wt% Al_0.5_CoCrFeNi_2_ compacted by SPS
AZ91–SiC	AZ91-10 wt% SiC compacted by SPS

**Table 2:** Composition of as-received HEA and different areas marked in AZ91–HEA and AZ91–SiC (at%).

Sample	Area	Element									
		Mg	Al	Mn	Zn	Co	Cr	Fe	Ni	Si	C
HEA	-	-	8.64	-	-	18.15	18.34	18.72	36.15	-	-
AZ91	A	90.91	8.76	0.13	0.21	-	-	-	-	-	-
AZ91	B	80.11	19.56	0.10	0.13	-	-	-	-	-	-
AZ91–HEA	A	91.45	8.13	0.10	0.31	-	-	-	-	-	-
AZ91–HEA	B	-	8.87	-	-	18.16	18.11	18.99	35.87	-	-
AZ91–SiC	A	91.36	8.21	0.13	0.30	-	-	-	-	-	-
AZ91–SiC	B	-	-	-	-	-	-	-	-	47.09	52.91

**Table 3:** Mechanical properties of the AZ91 alloy, AZ91–HEA and AZ91–SiC composites (Calculation of C.Y.S is given in Tables S1 and S2 in the Supplementary Materials).

Sample	Hardness (HV)	C.Y.S (MPa)	Failure Strain (%)
AZ91	93 ± 2	178 ± 4	12.2 ± 0.3
AZ91–HEA	138 ± 2	209 ± 8	13.7 ± 0.5
AZ91–SiC	123 ± 8	204 ± 10	12.0 ± 0.4

**Table 4:** Contribution of different strengthening mechanisms to increased yield strength of AZ91 composites.

Sample	Mechanism	Value (MPa)
AZ91–HEA	Thermal mismatch	10.0
AZ91–HEA	Grain boundary	1.0
AZ91–HEA	Load transfer	3.0
AZ91–HEA	Orowan	0.1
AZ91–HEA	Solid solution	0.3
AZ91–SiC	Thermal mismatch	9.9
AZ91–SiC	Grain boundary	4.0
AZ91–SiC	Load transfer	6.0
AZ91–SiC	Orowan	0.1
AZ91–SiC	Solid solution	0.1

## Data Availability

The authors confirm that the data supporting the findings of this study are available within the article.

## Supplementary Materials

The following are available online at https://www.mdpi.com/article/10.3390/ma14216520/s1, Figure S1: Enlarged section of engineering compressive stress-strain curves of AZ91-HEA composite, Figure S2: Enlarged section of engineering compressive stress-strain curves of AZ91-SiC composite, Table S1: Compressive yield strength (C.Y.S) of AZ91-HEA composite calculated using stress and strain curves shown in Figure S1, Table S2: Compressive yield strength (C.Y.S) of AZ91-SiC composite calculated using stress and strain curves shown in Figure S2.
