# Supplementary material for: Al0.5CoCrFeNi2 High Entropy Alloy Particle Reinforced AZ91 Magnesium Alloy-Based Composite Processed by Spark Plasma Sintering
Source: Materials (Basel). 2021 Oct 29;14(21):6520. doi: 10.3390/ma14216520 (PMC8585362; doi:10.3390/ma14216520)
Supplement: Supplementary file 1 [file materials-14-06520-s001.zip › materials-1409083-supplementary.pdf]

materials

MDPI

Supplementary Materials

$Al_{0.5}CoCrFeNi_2$ High Entropy Alloy Particle Reinforced AZ91 Magnesium Alloy-Based Composite Processed by Spark Plasma Sintering

Chun Chiu * and Hsun-Hsiang Chang

Department of Mechanical Engineering, National Taiwan University of Science and Technology, Taipei 106335, Taiwan; m10703517@mail.ntust.edu.tw
* Correspondence: cchiu@mail.ntst.edu.tw

Citation: Chiu, C.; Chang, H.-H. $Al_{0.5}CoCrFeNi_2$ High Entropy Alloy Particle Reinforced AZ91 Magnesium Alloy-Based Composite Processed by Spark Plasma Sintering. *Materials* **2021**, *14*, 6520. https://doi.org/10.3390/ma14216520

Academic Editor: Sergey V. Zherebtsov

Received: 20 September 2021
Accepted: 26 October 2021
Published: 29 October 2021

Publisher's Note: MDPI stays neutral with regard to jurisdictional claims in published maps and institutional affiliations.

Copyright: © 2021 by the authors. Licensee MDPI, Basel, Switzerland. This article is an open access article distributed under the terms and conditions of the Creative Commons Attribution (CC BY) license (http://creativecommons.org/licenses/by/4.0/).

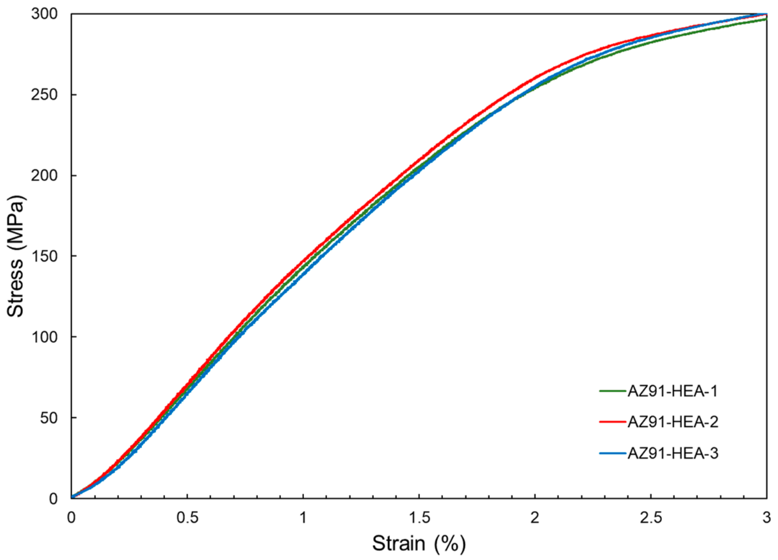

Figure S1. Enlarged section of engineering compressive stress-strain curves of AZ91-HEA composite.

Table S1. Compressive yield strength (C.Y.S) of AZ91-HEA composite calculated using stress and strain curves shown in Figure S1.

Test	1	2	3	Average ± STDEV
C.Y.S (MPa)	208	217	201	209 ± 8

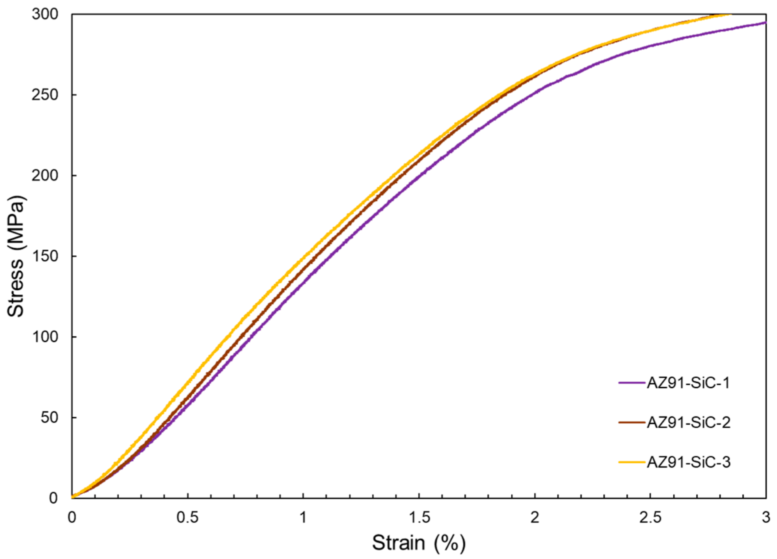

Figure S2. Enlarged section of engineering compressive stress-strain curves of AZ91-SiC composite.

Table S2. Compressive yield strength (C.Y.S) of AZ91-SiC composite calculated using stress and strain curves shown in Figure S2.

Test	1	2	3	Average ± STDEV
CYS (MPa)	194	204	214	204 ± 10
